# Oxidation-Based Continuous Laser Writing in Vertical Nano-Crystalline Graphite Thin Films

**DOI:** 10.1038/srep26224

**Published:** 2016-05-19

**Authors:** Loïc Loisel, Ileana Florea, Costel-Sorin Cojocaru, Beng Kang Tay, Bérengère Lebental

**Affiliations:** 1 CINTRA CNRS/NTU/Thalès, UMI 3288, 50 Nanyang Drive, Singapore; 2 School of Electrical and Electronics Engineering, Nanyang Technological University, 50 Nanyang Avenue, Singapore; 3 LPICM, CNRS, Ecole Polytechnique, Université Paris Saclay, 91128, Palaiseau, France; 4 Université Paris-Est, IFSTTAR, 14-20 Bd Newton, Champs-sur-Marne, F-77447, Marne-la-Vallée, France

## Abstract

Nano and femtosecond laser writing are becoming very popular techniques for patterning carbon-based materials, as they are single-step processes enabling the drawing of complex shapes without photoresist. However, pulsed laser writing requires costly laser sources and is known to cause damages to the surrounding material. By comparison, continuous-wave lasers are cheap, stable and provide energy at a more moderate rate. Here, we show that a continuous-wave laser may be used to pattern vertical nano-crystalline graphite thin films with very few macroscale defects. Moreover, a spatially resolved study of the impact of the annealing to the crystalline structure and to the oxygen ingress in the film is provided: amorphization, matter removal and high oxygen content at the center of the beam; sp^2^ clustering and low oxygen content at its periphery. These data strongly suggest that amorphization and matter removal are controlled by carbon oxidation. The simultaneous occurrence of oxidation and amorphization results in a unique evolution of the Raman spectra as a function of annealing time, with a decrease of the I(D)/I(G) values but an upshift of the G peak frequency.

As silicon transistors can no longer follow Moore’s law^1^, carbon is considered one of the most promising material for replacing or complementing silicon-based electronics^2^,^3^. Therefore, it is of primary interest to master the fabrication of carbon-based electronic chips that can achieve the desired data computing, storing and transmitting functions. Though short-wavelength (13.5 nm) laser-based lithography will be one of the most critical processes for the mass fabrication of high-performance transistors^4^ at resolutions below 10 nm^5^, a wide range of applications of carbon materials do not require such small features (thin-film transistors^4^ or sensors).

For such applications, laser light may be used directly (without a mask) to draw patterns onto layers of carbon allotropes without using photoresist, thus ridding the fabrication process of a well-known contaminating agent for carbon-based electronics (*e.g.* contamination of graphene by photoresist^6^,^7^,^8^).

Though the capability to amorphize graphitic carbon *via* pulsed laser writing would also be of interest, for instance to draw insulating lines in graphitic carbon, or to fabricate heat and radiation-resistant optical memories^9^, no report on amorphization is presently available. Moreover, pulsed laser writing also leads to undesired surface degradations such as sputtering^10^ or phase explosion^11^,^12^,^13^ due to the very short duration of the pulses (several nanosecond to several femtoseconds), yielding large power densities. At such short timescales, the light-matter interactions enabling energy dissipation are difficult to predict and thus optimize^14^.

By comparison, the physics behind slow thermal annealing is more easily modeled^15^,^16^,^17^,^18^,^19^ and controlled. Because they enable the deposition of energy in a slow manner, continuous-wave (CW) lasers can be used to locally transform matter in a manner similar to thermal (oven) annealing^20^,^21^. CW lasers are also cheap. They are thus being considered as an alternative to pulsed lasers for direct writing.

Despite these various studies, the literature on CW laser annealing of carbon does not provide a clear description of the mechanisms behind these phase change and matter removal processes. It is not clear whether the observed graphitization and amorphization processes occur in the solid-state or after melting, or what are the etching mechanisms. Indeed, it is well known that during oven-based, slow annealing experiments in air^22^,^23^,^24^, oxygen reacts strongly with carbon at temperatures below the melting point, resulting in carbon etching. Oxidation is seldom if ever studied as a mechanism for structural and morphological changes during laser-annealing of carbon allotropes (see Supplementary Information S1 for an analysis of the literature on carbon-laser interactions).

However, these questions should be answered to fully exploit CW lasers as low-cost tools for direct writing on carbon thin films.

In this study, we find that three main processes occur during CW laser annealing of vertical nano-crystalline graphite (vnC-G) in ambient atmosphere: at low power densities, sp^2^ clusters form, leading to an increase in the electrical conductivity. At higher power densities, oxidation starts, leading to both a degradation of the crystalline order in the depth and a removal of matter at the surface. We provide clear evidence that oxidation occurs during the CW-laser annealing of carbon, as we observe a strong correlation between the in-depth amorphization process and the spatial repartition of oxygen as measured by energy dispersive X-ray spectroscopy (EDX).

## Results and Discussion

### Crystalline structure of pristine vertical nano-crystalline graphite

**Figure 1:**
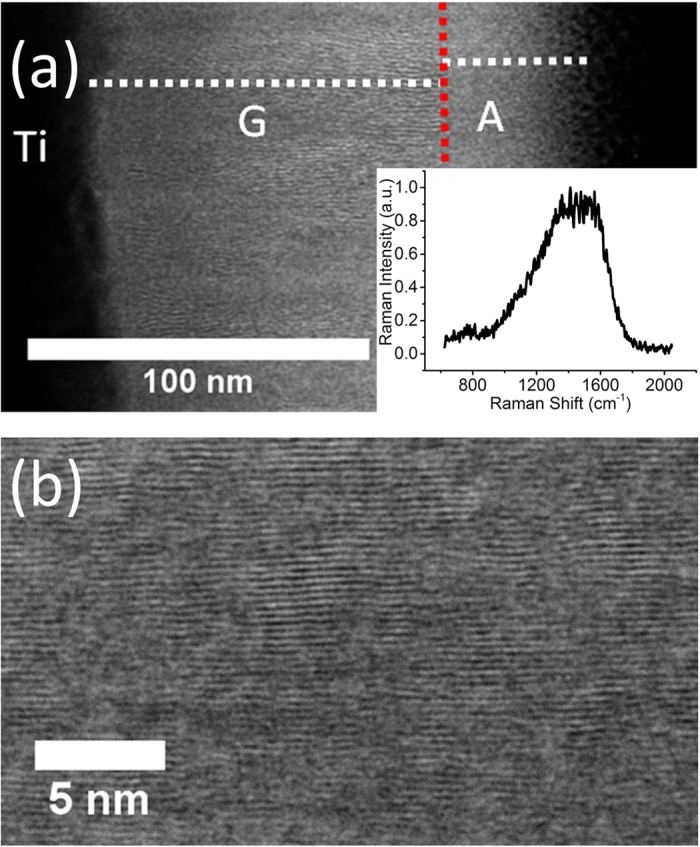
TEM characterization of pristine vnC-G. (**a**) Dark-field STEM micrograph showing two sublayers within the un-annealed v-ncG film: a 40 nm-thick amorphous layer (“A”) on top of a 100 nm-thick graphitic layer (“G”). *Inset*: Raman spectrum of pristine vnC-G. (**b**) HR-STEM Dark-Field micrograph on a zoomed area from the graphitic “G” layer illustrating the parallel graphitic planes.

Figure 1(a) presents a dark-field high-resolution scanning transmission electron microscopy (HR-STEM) micrograph of the as-deposited carbon film. The carbon layer is ≈140 nm thin, and comprises two sublayers: a graphitic layer of thickness ≈100 nm directly on top of the Ti layer (labeled “G” for graphitic) and, resting on it, an amorphous layer of thickness ≈40 nm (labeled “A” for amorphous). “G” consists of neighboring amorphous and crystalline regions, with graphite crystals as large as 50 nm being frequently observed (Fig. 1(a)), with a measured interplanar distance of ≈0.33 nm (Fig. 1(b)). It is comparable to the interplanar distance of pristine graphite (0.34 nm). On the other hand, no trace of crystalline content is observed in the top “A” layer.

Raman spectroscopy on the pristine vnC-G layer yields I(D)/I(G) ≈ 1.02 ± 0.03, x_G_ ≈ 1557 ± 2 cm^−1^ and full-width-at-half-maximum (FWHM) FWHM_G_ ≈ 254 ± 6 cm^−1^. The large G peak FWHM suggests average crystal sizes much smaller than 2 nm^25^, which is consistent with the properties of the “A” layer observed by TEM. Considering the penetration depth of the Raman photons in carbon (≈38 nm), the “G” layer is not probed much (Supplementary Information S2). However, graphitic crystals deeper than 35 nm can still have an impact on Raman spectra due to the large cross section of graphitic carbon^20^. Moreover, X-ray photoelectron spectroscopy (XPS) data suggest that the as-deposited vnC-G film contains 3 to 4 atomic % of oxygen; high-resolution O1s spectra show that these oxygen atoms form ≈70% of C=O bonds and ≈30% of C-O bonds (Supplementary Information S3).

### Matter Removal and Amorphization at beam center

**Figure 2:**
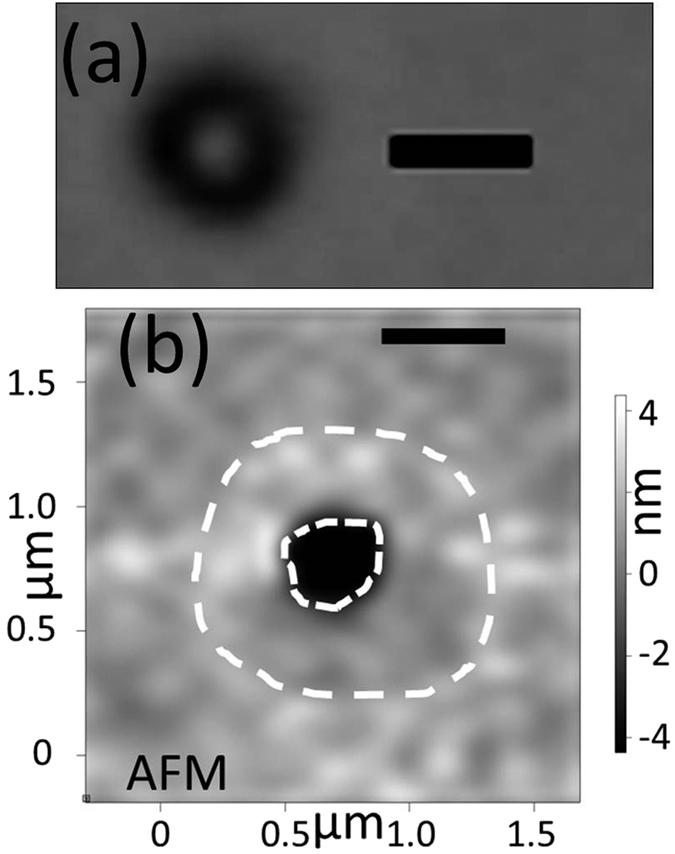
Morphology of laser-annealed vnC-G. (**a**) Optical image (scale bar 1 μm) of a laser-annealed region with energy density 5.9.10^6^ J. mm^−2^. (**b**) AFM 2D height image showing a crater (black surrounded by a white dashed line) and a surrounding bulge (marked with a white dashed line) of a laser-annealed region with energy density 1.7.10^7^ J. mm^−2^.

After characterization of the pristine film by TEM, Raman and XPS, we laser-anneal in air specific locations for varying durations and at different powers. After this process, locations that are annealed for the shortest durations feature single clear circular spots. With increasing durations and/or powers, a dark spot appears, and then extends outward with duration and power, until a bright inner disk appears at the center (Fig. 2(a)) (powers are varied between 8 and 22 mW and annealing durations between 1 s and 35 minutes).

**Figure 3:**
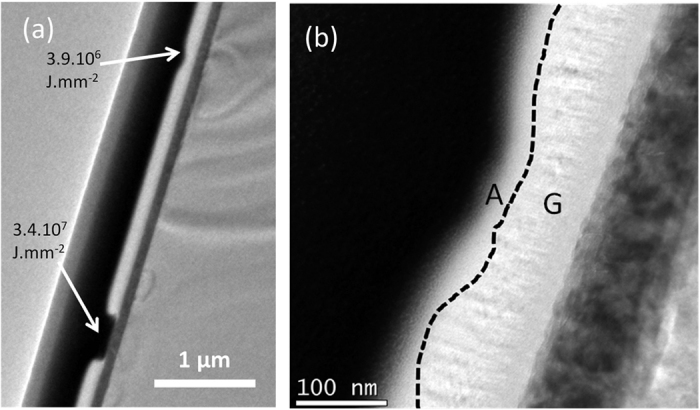
Cross-sectional TEM micrographs of laser-annealed vnC-G. (**a**) Cross-sectional TEM micrograph showing the two craters created by laser-annealing corresponding to two different power densities and durations. (**b**) TEM micrograph showing the structure of the crater created by annealing with energy density 3.9.10^6^ J.mm^−2^. The two “G” and “A” sublayers are visible.

AFM and TEM imaging reveal the topology of these spots: they feature a center crater surrounded by a slight bulge (Fig. 2(b)). The depth and diameter of the craters both increase with annealing power and/or duration, clearly indicating that matter removal occurs. The increase in depth stops when the Ti layer is reached (Fig. 3(a), 3.4.107 J.mm^−2^). The high reflectivity of the metallic Ti layer explains the bright spot observed optically for high power or long duration exposure.

TEM images (Fig. 3(b)) show that, as the annealing energy increases and the matter loss progresses, the thickness of the “G” (graphitic) layer decreases while the thickness of the “A” (amorphous) layer remains roughly constant, so that overall the material becomes more amorphous. While CW lasers have been reported as efficient tools to turn amorphous carbon into graphitic carbon^20^,^21^,^26^, their use as a tool to amorphize carbon has been reported only once^20^, with minor discussion on the underlying mechanism.

To understand more finely the impact of laser annealing on the crystalline structure of the top of the carbon layer, we carry out *in-situ* Raman spectroscopy during laser annealing. The spectra are acquired directly from the back-scattered signal from the annealing laser. As each acquisition takes only 15 s, the acquisition time is small as compared to the timescale of the *in-situ* experiment (from 300 s to 2,100 s).

**Figure 4:**
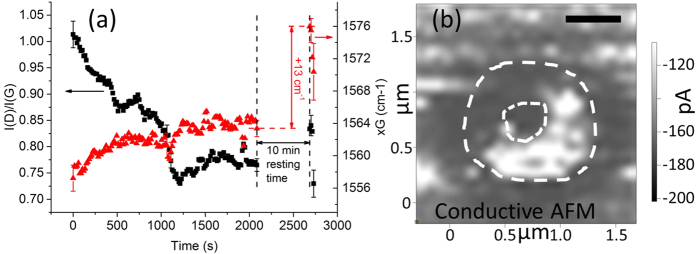
Raman and c-AFM data suggest that sp^2^ clustering takes place around the crater. (**a**) Evolution of the I(D)/I(G) ratio (black squares) and G peak position values (red triangles) as a function of time for a film annealed at 16 kW.mm^−2^. The last four spectra (after 10 min resting time) were taken with much lower Raman power densities. (**b**) (1.7.10^7^ J.mm^−2^) c-AFM image of a laser-annealed crater showing that the highest conduction occurs at the location of the bulge surrounding the hole.

Representative results of the *in-situ* study are displayed in Fig. 4(a). It is found that, until 1,080 s, the I(D)/I(G) ratio decreases, while the G peak width tends to increase slightly (Supplementary Information S4): both suggest a decrease of the average crystal size^25^.

### Graphitization at the periphery of the beam

At 1,080 s, a strong increase in the Rayleigh (elastic) scattering signal is observed (Supplementary Information S5). It is attributed to the full removal of the carbon layer that uncovers the highly reflective titanium film. It is consistent with the optical images of the spots showing a bright center. From this time onward, the carbon Raman scattering signal is thus attributed to the periphery of the beam. At 1,080 s also, the G peak width drops suddenly to reach value much lower than the pristine material (Supplementary Information S4). This strongly suggests that the periphery of the hole formed by the annealing has been graphitized compared to the pristine materials^27^.

Let us note that the minimum value for I(D)/I(G), and the subsequent slight rise consistent with graphitization, are reached at 1,215 s only. It suggests that between 1,080 s and 1,215 s, some interplay occurs between the signal coming from the bottom “G” and top “A” layer of the film.

To support the periphery graphitization hypothesis, conductive AFM (c-AFM) experiments are carried out. Data show that the circular bulge surrounding the crater is much more conductive than the pristine vnC-G film (Fig. 4(b)), thus suggesting it is more graphitic than the crater. The topology of the area, forming a bulge, is also consistent with graphitization, as the density of graphitized carbon (2.09–2.23 g/cm^3^) is known to be lower than the density of amorphous carbon. The same property has also been observed for the filtered-cathodic-vacuum-arc (FCVA) deposited carbon we are studying here^28^.

The enhanced graphitization at beam periphery, strongly different from the amorphization observed at the center, is explained by the lower power of the beam at its periphery (Supplementary Information S6). Indeed, low power CW annealing, as well as low power thermal annealing, are known to result in graphitization^15^,^16^,^17^,^18^,^19^,^20^,^21^,^26^.

### Post-annealing re-graphitization

After 10 minutes of air-cooling (Fig. 4(a)), it is also found that the I(D)/I(G) increases. Unlike the G peak frequency known to depend on the temperature^29^,^30^,^31^, this ratio value does not depend on the temperature. This increase suggests that re-graphitization takes place during the cooling phase, similarly to what happens during low temperature thermal annealing^15^,^16^,^17^,^19^. Though here, the final material is still more amorphous than the initial material, re-graphitization during cooling may partially explain why very few studies have been able to observe laser-induced amorphization of carbon thin films.

### Amorphization and matter loss by oxidation

During the first 1,080 s, while the I(D)/I(G) values and the G peak FWHM values in the Raman spectra are consistent with amorphization at the beam center, the G peak frequency increases significantly (Fig. 4(a)). It is unexpected from the usual framework for Raman spectroscopy interpretation^25^,^27^, as it should decrease with amorphization. However, other factors than the crystalline content have been reported to impact the G peak frequency, such as the temperature^29^,^30^,^31^ or hole doping^32^.

Here, the temperature rise due to the annealing process cannot explain this G peak *upshift*, as a *downshift* of the peak frequency by 0.013 cm^−1^/K is actually expected with increasing temperature^29^,^30^,^31^.

**Figure 5:**
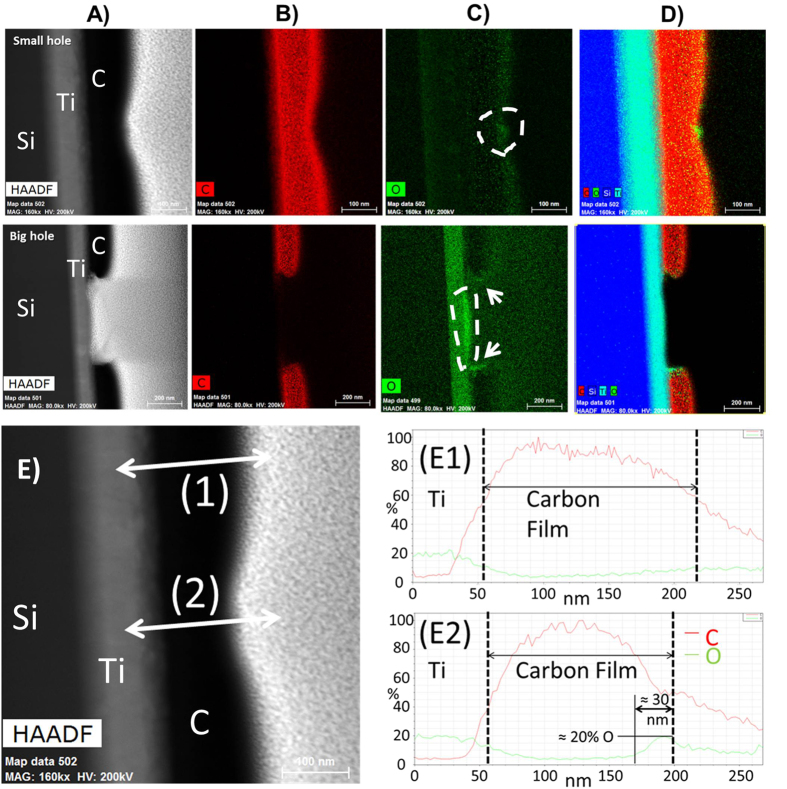
EDX chemical mappings on two craters to study the oxygen concentration. Top row: 3.9.10^6^.mm^−2^. Bottom row: 3.4.10^7^.mm^−2^. (**A**) STEM micrographs. (**B**) Carbon elemental map. (**C**) Oxygen elemental map. (**D**) Relative map showing the distribution of each element with the sample with carbon in red, silicon in dark blue, titanium in light blue and oxygen in green. (**E**) EDX-STEM line scan analysis on two different positions, far and close to the beam center, for the spot annealed with an energy density of 3.9.10^6^ J.mm^−2^ (E1) Carbon (in red) and oxygen (in green) concentrations recorded along the white arrow labeled (1). (E2) Carbon (in red) and oxygen (in green) concentrations recorded along the white arrow labeled (2).

Hole doping is another factor known to cause an upshift of the G peak^32^. To verify its relevance here, a chemical characterization of the post-annealing material is carried out using energy dispersive spectroscopy (EDX) coupled with the scanning imaging mode of the electron microscope. Figure 5 presents 2D EDX-STEM chemical maps obtained for the carbon Kα-ionization edge (B) and oxygen Kα-ionization edge (C) on two craters previously characterized by TEM (Fig. 3(a)).

Oxygen appears to be present in larger quantities at laser-annealed locations than in the pristine material. As oxygen is a strong hole dopant for carbon^33^ materials, it explains the G peak position rise observed in the Raman data. The G peak upshift still remains moderate because while the hole doping is progressing, the temperature in the material is also rising.

Moreover, the highest oxygen concentrations are found at the highest power locations, either on carbon or on titanium (when the carbon layer is fully removed). The oxidation of titanium explains why no drastic conductivity rise is observed when the c-AFM tip is in direct contact with it at the center of the inner disk (Fig. 4(b)), as titanium oxide is an insulator.

Line scan analyses (Fig. 5(E)) show that the quantity of oxygen atoms increases considerably beneath the surface of laser-annealed carbon (from ≈5% to ≈20%, see data on Fig. 5(E2)), down to ≈30 nm from the surface. These depths compare well to the amorphization depths observed by TEM (Fig. 3(b)). Overall, it suggests that the amorphization and the matter removal are strongly related to the presence of oxygen in the annealed carbon layer.

We propose that the strong ingress of oxygen is due to oxidation of the carbon layer. This hypothesis could not be proved directly, as no change in the C/O bonds ratio could actually be observed by XPS (Supplementary Information S3) due to the large size of the XPS beam (smallest diameter ≈100 μm) as compared to the laser-modified area (≈1 μm diameter).

To support the postulate that oxidation drives the amorphization and the matter loss, the temperature during annealing is estimated. The upshift of the G peak right after the annealing is measured to be ≈13 cm^−1^ (Fig. 4(a)), yielding the conclusion that the temperature reaches at most about 1,300 K during laser exposure^29^,^30^,^31^. This rather low temperature estimate is confirmed by the fact that the Ti layer is found to be mostly un-damaged (Fig. 3(a)), while the melting temperature of Ti is ≈1,941 K. The temperature of 1,300 K may actually be an overestimation, as some hole doping may keep occurring after the annealing is over, thus contributing to the upshift of the G peak.

This temperature range is way below the temperatures required for melting, sputtering or phase explosion of carbon (above 4,300 K^34^), other common explanations for matter removal during carbon-laser interactions. Additionally, the remaining matter does not display the major damages usually associated with processes occurring at the melting temperature: the roughness of the area surrounding the crater is found (by AFM characterization) to be below 1 nm.

On the other hand, the range of temperatures around 1,300 K is precisely in the range to enable carbon oxidation^23^, as is reported in oven-based thermal annealing experiments^22^,^23^,^24^. Moreover, oxidation is known to cause amorphization by doping (introduction of larger oxygen atoms into sp^2^ clusters leading to a loss of short and long range order^35^) and matter loss by formation of gaseous CO and CO_2_^22^,^36^, which is precisely what is observed at locations where the oxygen content is the largest.

**Figure 6:**
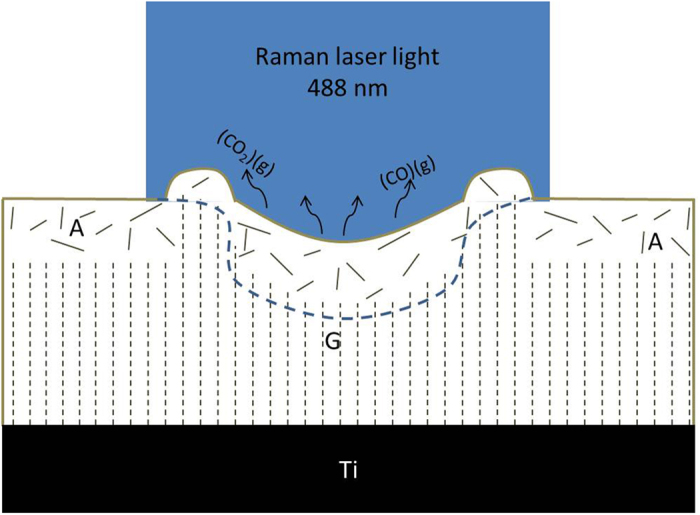
Schematic cross-section of the carbon thin film, composed of the two layers “A” and “G”, during CW laser annealing. It shows the matter loss process by oxidation in the center (formation of gas (CO) and (CO_2_) molecules), the amorphization process, the sp^2^ clustering leading to a change in density. An arbitrary estimate of the region where most of the Raman information is collected is drawn in dashed blue. It highlights the importance of estimating the penetration depths of the photons when the characterized material is in-homogeneous along its depth.

These elements all support the fact that the high oxygen content at annealed location is due to oxidation of the carbon layer, causing amorphization and matter loss. Figure 6 summarizes the phenomena occurring during laser annealing based on this interpretation.

### Application to continuous-wave laser writing

**Figure 7:**
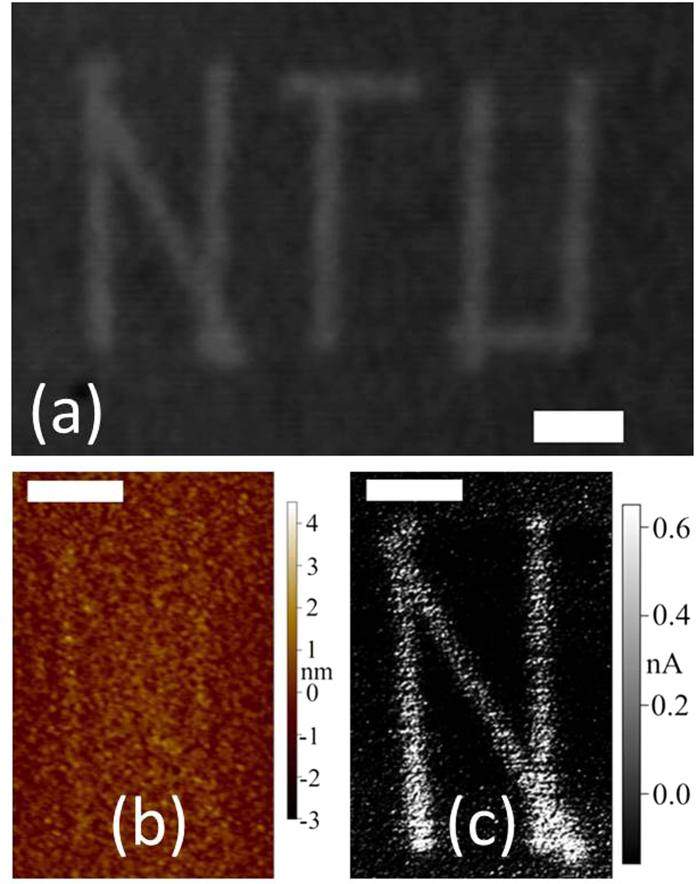
Example of a pattern drawn on vnC-G using a CW 488 nm laser beam. (**a**) Optical image. (**b**) AFM image (contact mode) of the “N” showing the height of the features. (**c**) c-AFM image of the “N” showing the conductivity of the features. Scale bars: 2 μm.

Based on this analysis of the mechanisms occurring during CW laser annealing, we demonstrate the writing capability of a CW laser. Figure 7 shows the acronym “NTU” drawn on a sample by applying a power density of 1.2 kW.mm^−2^ for 1 s at each spot. The shape was achieved with a total of 154 s exposure by using the line mapping capabilities of the Raman WITec system. This relatively low level of power and duration is expected to provide fine patterns with slightly graphitized and/or oxidized carbon. Figure 7(b) shows that the surface morphology is only slightly modified by the writing. On the contrary, the electrical conductivity (Fig. 7(c)) is largely enhanced, suggesting the occurrence of sp^2^ clustering. Though the time required for the patterning is rather long as compared to techniques based on nanosecond or femtosecond lasers, it can be achieved with relatively low-cost equipment and yields a material with a highly controlled surface state (roughness within the pattern and outside the pattern are both lower than 1 nm).

## Conclusion

We have studied the annealing of vnC-G via CW laser and demonstrated the potential of this technique for laser writing. CW laser writing results in amorphization and matter removal at the center of the beam, with an intensity controlled by the power density and the duration of exposure. On the contrary, in the periphery of the beam, where the exposure is less intense, sp^2^ clustering is detected. Spatially sensitive chemical analysis shows the strong presence of oxygen in the post-annealing material, suggesting that amorphization and matter removal are controlled by carbon oxidation. The simultaneous occurrence of amorphization and oxygen ingress, which appears to be characteristic of this process, results in a unique evolution of the Raman spectra, with a decrease of the I(D)/I(G) and an increase of the FWHM of the G peak, but an upshift of the G peak attributed to oxygen-induced hole doping. Altogether, as the matter removal process is quite slow, the resulting annealed matter features very few macroscale defects. As a consequence, CW laser writing on vnC-G results in very clean structures with low surface roughness.

## Methods

### Film deposition

A ≈100 nm thick Ti layer is deposited on a clean Si substrate using electron-beam evaporation. The carbon layer is then deposited onto the Ti layer using a FCVA^37^, with deposition time ≈3 min 58 s and vacuum pressure ≈5.10^−5 ^Torr. An arc current of 60 A is applied to the graphite target and an accelerating voltage of −300 V DC is applied to the substrate holder. The resulting film is 140 nm thick as evidenced by cross-sectional TEM.

XPS surface chemical analyses were carried out with a Thermo electron K-alpha spectrometer using a monochromatic Al-Kα X-ray source (1486.6 eV). The Thermo K-alpha spectrometer procedure was used to calibrate the spectrometer. It was verified using Cu and Au (Au 4f7/2 at 84.0 eV) samples following the ASTM-E-902-94 standard procedure. Acquisition parameters imposed in this study were the following: 200 μm spot size, 12 kV primary energy, 2.5 mA emission intensity, CAE 200 eV and 1 eV energy step size for survey spectra, and CAE 10 eV and 0.05 eV energy step size for high resolution spectra. Quantification was performed with the Thermo Fisher Scientific Avantage© data system. For some XPS analyses, a 10 s Ar+ sputtering, at 0.2 nm/s etching rate (equivalent rate/Ta_2_O_5_ standard), was performed to remove adventitious carbon from the top of the sample. The film composition homogeneity in depth was also verified after a second Ar+ sputtering: 20 s at 0.75 nm/s etching rate conditions (equivalent rate/Ta_2_O_5_ standard).

### Annealing and Raman characterization

WITec 488 nm and 532 nm lasers are used for both annealing and Raman spectroscopy. The laser is fed into the x100 objective of a confocal microscope leading to a ≈1 μm diameter spot at the proper working distance. The power density of the laser is measured with a THORLABS S121C photodiode connected to a THORLABS PM100D display. It is approximated by dividing the power by the area of a disk of 1 μm diameter. The laser energy spread follows a Gaussian distribution along the radius with maximum power density at the center of the beam (Supplementary Information S6). The surface of the vnC-G film is laser-annealed for varying durations (from 1 to 35 minutes) and power densities (from ≈13 to 28 kW.mm^−2^) at different locations of the film. During laser annealing, Raman spectra are collected every 15 s. Acquisition time varies between 300 and 2,100 s.

Typical Raman spectra of vnC-G consist of three broad peaks centered at ≈1060 cm^−1^, ≈1350 cm^−1^ and ≈1580 cm^−1^ called the T, D and G peak, respectively^25^,^38^. The T peak arises due to vibrations of the sp^3^ bonds, the G peak arises because of photon interactions with stretching vibrations of pairs of sp^2^ C bonds, while the D peak appears in presence of defective graphitic rings. The D to G peak intensity ratio I(D)/I(G) is used to determine the amount of sp^2^ crystalline content forming rings, and the size of sp^2^ crystals can be estimated from it, using either the Tuinstra and Koenig (T-K) equation^39^ or an equation derived by Ferrari *et al.*^27^, depending on the actual range of crystal sizes.

Using a program written in Scilab^40^, we fit the Raman with three Lorentzian functions for the T (centered at *ca.* 1060 cm^−1^), D (1350 cm^−1^) and G (1580 cm^−1^) peak. To estimate noise-related errors on the Raman spectra, we use the Bootstrap method (Supplementary Information S7): we find standard deviations values on I(D)/I(G) of ≈0.04 and x_G_ ≈ 4 cm^−1^. Some representative errors are calculated and the corresponding error bars are displayed on the figures.

### Post-annealing characterization

For the TEM/STEM analysis, cross-sections are first prepared on selected regions of the film using a FIB-Scios dual beam microscope. The HR-TEM and STEM-EDX chemical analyses are performed using a Titan-Themis electron microscope operating at 200 kV, equipped with a Cs probe corrector and a SuperX detector. For the STEM-EDX analysis, several 1D EDX spectra are recorded at various locations of the electron beam focused probe using a convergent angle α of about 25 mrad and a collection angle β of 30 mrad.

To obtain information on the surface morphology, we use an Asylum Research Cypher S AFM in tapping mode. We also characterize the through-film electrical properties in c-AFM mode by applying an electrical potential to the substrate and measuring the electrical current going through the tip and sample. For that method, we use Pt-coated silicon tips.

## Additional Information

**How to cite this article**: Loisel, L. *et al.* Oxidation-Based Continuous Laser Writing in Vertical Nano-Crystalline Graphite Thin Films. *Sci. Rep.* **6**, 26224; doi: 10.1038/srep26224 (2016).

## Supplementary Material

Supplementary Information
