# Supplementary material for: Oxidation-Based Continuous Laser Writing in Vertical Nano-Crystalline Graphite Thin Films
Source: Sci Rep. 2016 May 19;6:26224. doi: 10.1038/srep26224 (PMC4872136; doi:10.1038/srep26224)
Supplement: Supplementary Information [file srep26224-s1.pdf]

Supplementary Information - Oxidation-Based Continuous Laser Writing in Vertical Nano-Crystalline Graphite Thin Films

Loïc Loisel, Ileana Florea, Costel-Sorin Cojocaru, Beng Kang Tay, Bérengère Lebental

S1 – Analysis of the literature on laser-carbon interactions.

Supplementary Table S1 provides a detailed review of the literature on laser-carbon interactions, showing the general goals of the reported studies. One observes that oxidation upon laser annealing is a seldom treated issue (7 papers over 42, over the course of 30 years), and mostly studied as a consequence of nanosecond laser pulses. Ref.[1] mentions oxidation during CW laser annealing, but does not discuss or characterize the actual oxidation mechanism.

Among these 42 references, none of them studies the spatial repartition of oxygen in the annealed material. Information on the type of bonds that carbon forms with oxygen (*e.g.* C=O, C-O…) is often provided by X-ray photoelectron spectrometry (XPS), but only for the pristine material[2] or for large samples of modified material[3,4], as the XPS spatial resolution of standard laboratory systems is often limited to spot sizes ≈ 100 µm.

Supplementary Table S1 - Literature on laser-carbon interactions with emphasis on the analysis of oxidation mechanisms.

Ref	Year	Base material	Laser properties	XPS (type of C/O bonds)	Mechanism studied	Oxidation (proof or impact)
5	1984	Graphite	Ruby<br>30 ns<br>694.3 nm<br>Spot size:<br>6 mm	No	Measurement of melting point and latent heat of	No

					fusion	
6	1985	Graphite	Ruby<br>30 ns<br>694.3 nm	No	Development of a model of heat transport during annealing	No
7	1986	Graphite	20 ps<br>532 nm	No	Study of melting and liquid phase	No
8	1989	Graphite	90 fs<br>620 nm<br>Spot size:<br>30 μm	No	Study of transient liquid phase	No
9	1990	Graphite	30 ns-694.3 nm<br>20 ps-532 nm<br>Spot size:<br>3-5 mm	No	Study of damages	No
10	1992	Graphite and diamond	90 fs<br>620 nm	No	Study of the liquid phase	No
11	1992	Hydrogenated amorphous carbon	CW<br>514.5 nm<br>Spot size:<br>2 μm	No	Structural changes	No
12	1995	Glassy carbon	25 ns<br>694.3 nm	No	Structural changes	No
13	1997	Diamond-like composite a-(C:H/Si:O)	CW<br>515.5 nm<br>Spot size:<br>2 μm	No	Structural changes	No
14	1998	Hydrogenated amorphous carbon	CW<br>514.5 nm<br>(ar-ion)<br>632.8 nm<br>(He-Ne)	No	Structural changes	No
15	2000	Graphite	Arf and Krf<br>193-248 nm<br>15 ns	No	Structural changes and ablation	Yes (literature)
16	2001	Graphite	Nd :YAG<br>532 nm<br>1064 nm<br>50 ns	No	Fabrication of single wall carbon nanotubes	No
17	2002	Single wall nanotubes	He-Ne<br>632.8 nm<br>Ar-ion 488 nm	No	Structural changes	No
18	2002	Doped carbon rod	Nd-YAG<br>1064 nm<br>Spot size:	No	Fabrication of single wall	No

			6 mm		carbon nanotubes	
19	2008	Nanotube thin film	Nd:YAG 532 nm 6 ns	No	Structural changes	Yes (cleaning of amorphous /defective CNTs during annealing)
20	2008	Amorphous carbon	KrF 248 nm 25 ns Spot size: 8*3 mm^2	No	Structural changes	No
21	2009	Graphite	Ti:sapphire 800 nm 100 fs	No	Ablation	No
22	2009	Amorphous carbon	Nd:YAG 355 nm Spot size: 12 mm^2 532 nm Spot size: 30 mm^2	No	Structural changes	No
4	2010	Graphite oxide	ND:YAG 1064 nm 532 nm 7 ns 355 nm	Yes	Reduction	No
23	2010	Graphite oxide	CW 532 nm Nd:YAG 532 nm 355 nm 9 ns	No	Reduction	No
24	2010	Graphene oxide	790 nm 120 fs	Yes (C-O, C=O, C-C)	Reduction	No
25	2011	Carbon film	Excimer 248 nm 23 ns	No	Annealing	No
26	2011	Graphite oxide	Mercury lamp (UV)	No	Reduction	No

27	2011	Multi-layer graphene	248 nm<br>20 ns	No	Ablation	Yes (avoided by working in vacuum)
1	2011	Multi-layer graphene	CW<br>532 nm<br>0.9-10 µm/s	No	Oxidative burning of top layers	Yes (Raman G-band shift)
28	2012	Carbon film	KrF<br>248 nm<br>23 ns	No	Annealing	No
29	2012	Graphite	Nd-YAG<br>1064 nm<br>10 ns	Yes (of laser-generated graphene)	Pulsed laser deposition of graphene	Yes (XPS spectra)
30	2012	Graphene oxide	532 nm<br>Spot size:<br>1 µm	Yes	reduction	No
3	2013	Graphite oxide	Nd:YAG<br>532 nm<br>10 ns	Yes (C-O, C=O, C-C)	Reduction	No
31	2013	Graphene oxide	KrF<br>248 nm<br>20 ns<br>Spot size:<br>10x10 μm^2	No	Reduction	No
32	2013	Graphite oxide	KrF<br>248 nm<br>25 ns<br>Spot size:<br>1.8*1.98 mm^2	Yes	Reduction	No
33	2013	Graphene oxide	KrF<br>248 nm<br>20 ns<br>Spot size:<br>20*10 mm^2	Yes (C-O, C=O, C-C)	Reduction	No
2	2013	Graphene	266 and 532 nm<br>5 ns<br>Spot size:<br>30µm*10µm	Yes (untreated area only)	Ablation	Yes (fluorescence)
34	2014	Graphene oxide	800 nm<br>120 fs	Yes (C-O, C=O, C-C)	Reduction	No
35	2014	Graphene oxide	Nd:YAG<br>532 nm<br>5 ns	Yes (C-O, O-C=O, C-C)	Reduction	No

			Spot size: 28 mm^2			
36	2014	Reduced graphene oxide	1030 nm 170 fs Spot size: ≈ 100 µm	No	Fabrication of field emitters	No
37	2014	Graphene	343 nm 550 fs Spot size: 400 nm	No	Ablation	Yes (Raman D-band)
38	2014	Multi-layer graphene	Ti:sapphire 840 nm 150 fs	No	Formation of periodic structures	No
39	2014	Diamond like carbon	KrF 248 nm 20 ns	No	Pulsed laser deposition of few-layer graphene	No
40	2014	Graphite	Nd:YAG 2nd harmonic	No	Pulsed laser deposition of few-layer graphene	No
41	2014	Graphite	KrF 248 nm 25 ns	No	Pulsed laser deposition of few-layer graphene	No
42	2015	Graphene oxide	415 nm 10 ns	Yes (C=C, C-O-C, C(O)-OH)	Fabrication of nanostructures + reduction	No

S2 - Calculation of the penetration depth of 488 nm photons into amorphous carbon

Following the standard framework, to estimate the penetration depth of photons into amorphous carbon, we use the Beer-Lambert law[43]:

$$I(\lambda, x) = I_0 e^{-\alpha(\lambda)x}$$

where I is the light intensity at depth x, I_0 is the incident light intensity and α is the attenuation constant. Then, the penetration depth is defined as:

$$\delta = \frac{1}{\alpha}$$

It is the depth at which the remaining electromagnetic power P equals 13% of its initial value P_0 (87% of the power has been absorbed).

The attenuation constant α can be calculated from the expression:

$$\alpha = 2\frac{\omega}{c} Im(n)$$

where ω is the light angular frequency (rad.s^{-1}), c is the speed of light and n is the complex index of refraction. The imaginary part of the complex index of refraction of amorphous carbon at the wavelength of interest (488 nm) is provided in Stagg *et al.*[44]

Supplementary Table S2 - Values of the imaginary part of the complex index of refraction of amorphous carbon Im(n), the attenuation constant α and the penetration depth δ as a function of the temperature. Values of the imaginary part of the index of refraction were obtained from the literature[44].

	**25°C**	**200°C**	**400°C**	**600°C**
**Im(n)**	1.003	1.635	1.001	1.001
**α (m^{-1})**	$2.58.10^7$	$4.21.10^7$	$2.58.10^7$	$2.58.10^7$
**δ (nm)**	38	24	38	38

So for temperatures ranging from 25 to 600°C, the penetration depth remains lower than 38 nm.

S3 – X-ray photo electron spectroscopy

X-ray photo electron spectroscopy spectra shown in Supplementary Figure S1 are obtained with a spot size of 200 µm. Spectra were acquired after 10 to 20 s abrasion time to remove superficial contamination (details on the XPS systems and the abrasion are provided in the experimental section of the main manuscript). XPS data suggest that the as-deposited film contain 3 to 4 atomic % of oxygen; high resolution O1s spectra show that these oxygen atoms form ≈ 70% of C=O bonds and ≈ 30% of C-O bonds (Supplementary Figure S1 (a)).

No significant difference between the annealed and the un-annealed material is detected (Supplementary Figure S1 (b)) by XPS due to the relatively large size of the XPS beam (200 µm) as compared to the CW laser spot (1 µm).

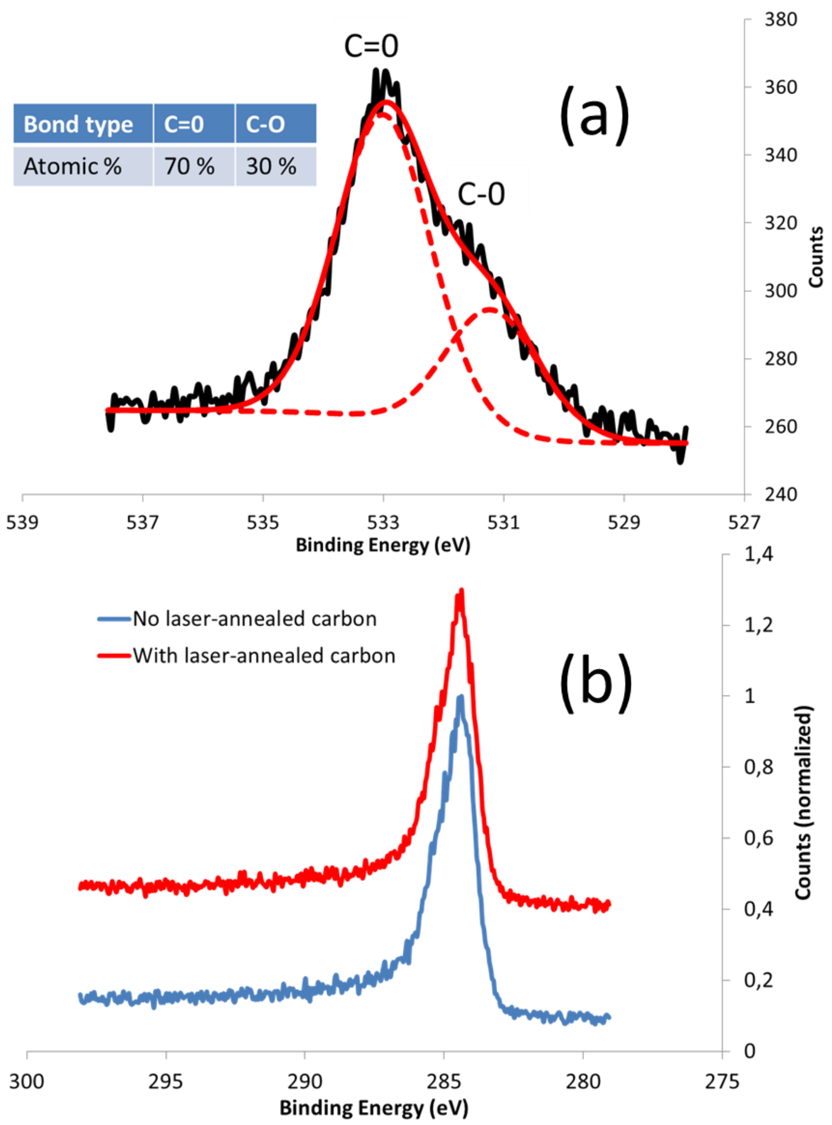

Supplementary Figure S1 - High-resolution XPS spectra of vnC-G with and without laser-annealed patterns. (a) O1s spectrum of un-annealed vnC-G obtained after a 20s abrasion step. (b) C1s spectra of un-annealed vnC-G (bottom) and of an area containing laser annealing vnC-G (top). Both spectra were obtained after a 10s abrasion step.

S4 - Evolution of the G peak width as a function of time

The width of the G peak (Supplementary Figure S2) features a strong drop at 1,080s, suggesting a sudden increase in the fraction of more graphitic matter. This occurs at the same time as a sudden increase in the amount of reflected light (Supplementary Figure S3), which we assign to the removal of carbon at the center of the crater (uncovering of the Ti layer). Hence, this result suggests that the remaining carbon (on the sides of the crater) is more graphitic than the carbon at the center.

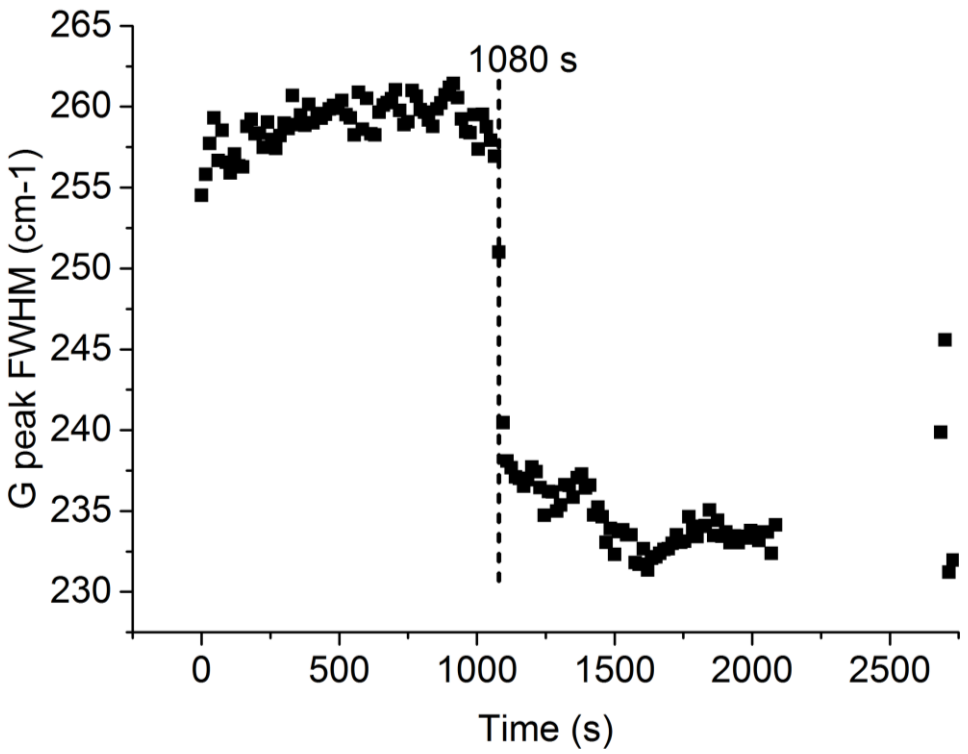

Supplementary Figure S2 – Evolution of the G peak width as a function of time.

S5 - Evolution of the Rayleigh scattering maximum as a function of time

The TEM images show that in some cases, matter is removed until the Ti layer is directly exposed to the laser beam. As Ti is highly reflective, we expect to observe a jump in the reflected light when Ti first gets directly exposed to the light. The reflected light is monitored qualitatively by measuring the intensity of the Rayleigh scattering of the Raman signal, namely the intensity of the peak centered at 0 cm^{-1} in terms of Raman shift (elastic process). In Supplementary Figure S3, we plot the values of the maximum of the Rayleigh peak as a function of time. It oscillates until 1,080 s, at which time the spectrometer becomes saturated. This result supports the fact that the Ti layer is first fully exposed at time = 1,080 s. The oscillation in elastically reflected light before 1,080 s may be due to changes in the depth of the hole, which in turn change the amount of light that is trapped in the hole.

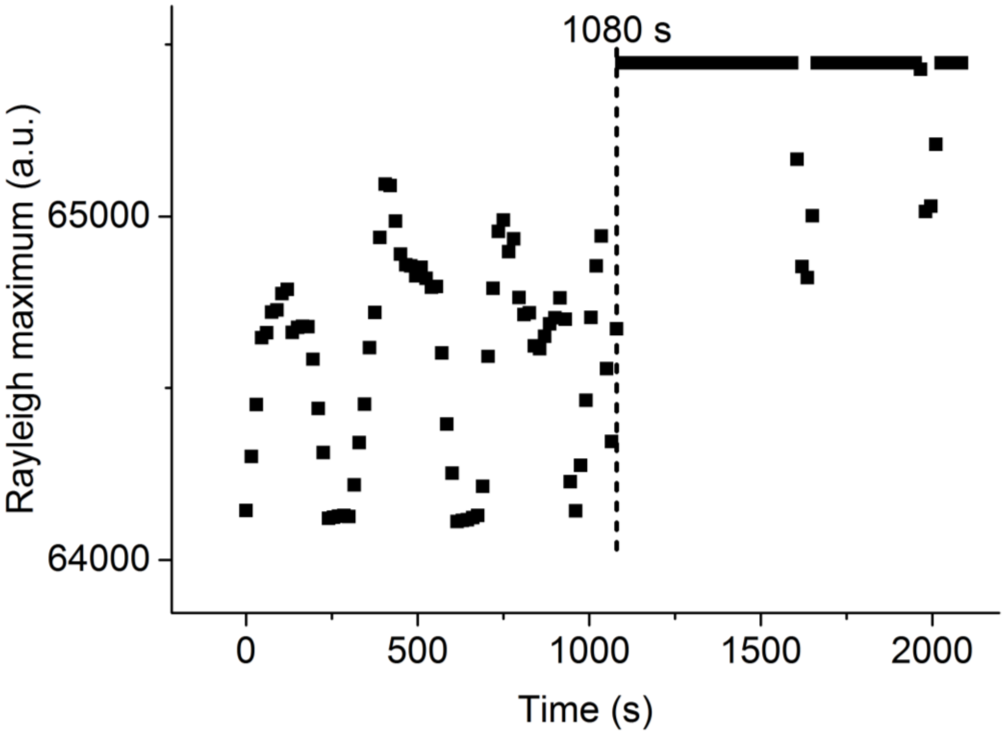

Supplementary Figure S3 – Evolution of the maximum values of the Rayleigh peak as a function of time.

S6 - Beam profile

We use a CMOS camera to obtain the power distribution of the beam of the 488 nm laser (Supplementary Figure S4). It is a simple Gaussian beam, with a maximum power at the center.

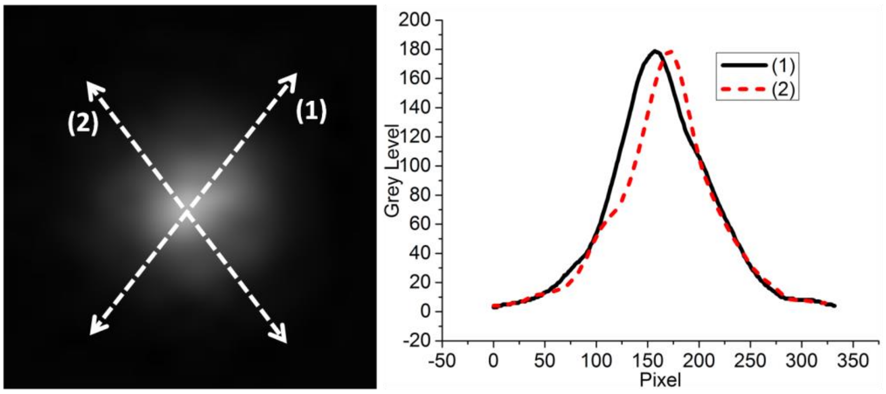

Supplementary Figure S4 - Beam profile of the 488 nm laser obtained with a THORLABS DCC1545M CMOS camera.

S7 - Fitting the Raman spectra and extracting data on the standard errors with Scilab

We fit Raman spectra with three Lorentzians for the T, D and G peaks and add a baseline. The corresponding Scilab[45] code is:

```
function y=lorentz1(x, p)      // Sum of three Lorentzian peaks and a horizontal baseline
a=p(1)  // Baseline
b=p(2)   // Amplitude Lorentz 1
c=p(3)   // x0 Lorentz 1   D peak
d=p(4)   // Tau Lorentz 1
e=p(5)   // Amplitude Lorentz 2
f=p(6)   // x0 Lorentz 2   G peak
g=p(7)   // Tau Lorentz2
h=p(8)  // Amplitude Lorentz 3
i=p(9)   // x0 Lorentz 3   T peak
j=p(10)  // Tau Lorentz 3

// Definition of the function
y=((x-c)^(2)+0.25*d^2)^(-1)*(0.5*b*d)+d+((x-f)^(2)+0.25*g^2)^(-1)*(0.5*e*g) +((x-i)^(2)+0.25*j^2)^(-1)*(0.5*h*j)
endfunction

function e=G(p, z)    // Function to calculate the error at each iteration
x=z(1)
y=z(2)
e=y-lorentz1(x,p)
endfunction

p0=[100;10;1350;200;10;1580;200;5;1060;200] // Initialization

[p,err]=datafit(G,Z,p0)    // Calculation of the p parameters (Least Square Method)
```

Then, we use the Bootstrap method to calculate random subsets of data from the initial data set. These subsets are then also fitted by three Lorentzians and a baseline, and the extracted

parameters (I(D)/I(G), xG, etc…) are all saved. Then, we calculate the standard error on each relevant parameter extracted for the initial subset and all the Bootstrap subsets. The method to generate a Bootstrap subset is given in the Scilab[45] code below:

```
taille=size(var1);                    // How many data points in the initial set (var1)?

bootnum=400;                          // Number of data subsets to generate (20)
datanum=taille(1);                    // Number of random indexes generated per subset (equal to the number of data points)
bootdata=zeros(datanum,2*bootnum);          // All the generated data will go into this matrix

for j=1:bootnum                       // For each data subset iteration

bootrand=round((datanum-1)*rand(datanum,1));    // Vector of random indexes between 0 and N-1
```

REFERENCES

1 Han, G. H. *et al.* Laser Thinning for Monolayer Graphene Formation: Heat Sink and Interference Effect. *ACS Nano* **5**, 263-268; doi:10.1021/nn1026438 (2011)

2 Kiisk, V., Kahro, T., Kozlova, J., Matisen, L. & Alles, H. Nanosecond laser treatment of graphene. *Appl. Surf. Sci.* **276**, 133-137; doi:10.1016/j.apsusc.2013.03.047 (2013)

3 Afshani, P., Attah, I., Moussa, S., Terner, J. & El-Shall, M. S. Hydrogen-Terminated Graphene by Laser Vaporization-Controlled Condensation of Graphite Oxide. Observation of Hydrogen-Capped Carbon Chains CnH–, CnH+, and CnH2+ (n = 2–30) in the Gas Phase. *J. Phys. Chem. C* **117**, 9485-9495; doi:10.1021/jp401318d (2013)

4 Abdelsayed, V. *et al.* Photothermal Deoxygenation of Graphite Oxide with Laser Excitation in Solution and Graphene-Aided Increase in Water Temperature. *J. Phys. Chem. Lett.* **1**, 2804-2809; doi:10.1021/jz1011143 (2010)

5 Venkatesan, T. *et al.* Measurement of Thermodynamic Parameters of Graphite by Pulsed-Laser Melting and Ion Channeling. *Phys. Rev. Lett.* **53**, 360-363; doi:10.1103/PhysRevLett.53.360 (1984)

6 Steinbeck, J., Braunstein, G., Dresselhaus, M., Venkatesan, T. & Jacobson, D. A model for pulsed laser melting of graphite. *J. Appl. Phys.* **58**, 4374-4382; doi:10.1063/1.335527 (1985)

7 Malvezzi, A. M., Bloembergen, N. & Huang, C. Y. Time-resolved picosecond optical measurements of laser-excited graphite. *Phys. Rev. Lett.* **57**, 146-149; doi:10.1103/PhysRevLett.57.146 (1986)

8 Reitze, D. H., Wang, X., Ahn, H. & Downer, M. Femtosecond laser melting of graphite. *Phys. Rev. B: Condens. Matter* **40**, 11986; doi:10.1103/PhysRevB.40.11986 (1989)

9 Speck, J. S., Steinbeck, J. & Dresselhaus, M. Microstructural studies of laser irradiated graphite surfaces. *J. Mater. Res.* **5**, 980-988; doi:10.1557/JMR.1990.0980 (1990)

10 Reitze, D. H., Ahn, H. & Downer, M. C. Optical properties of liquid carbon measured by femtosecond spectroscopy. *Phys. Rev. B: Condens. Matter* **45**, 2677-2693; doi:10.1103/PhysRevB.45.2677 (1992)

11 Bowden, M., Gardiner, D. J. & Southall, J. M. Raman analysis of laser annealed amorphous carbon films. *J. Appl. Phys.* **71**, 521-523; doi:10.1063/1.350691 (1992)

12 Vitali, G., Rossi, M., Terranova, M. L. & Sessa, V. Laser-induced structural modifications of glassy carbon surfaces. *J. Appl. Phys.* **77**, 4307-4311; doi:10.1063/1.359558 (1995)

13 Wan, J. Z., Pollak, F. H. & Dorfman, B. F. Micro Raman study of diamondlike atomic-scale composite films modified by continuous wave laser annealing. *J. Appl. Phys.* **81**, 6407-6414; doi:10.1063/1.364421 (1997)

14 Lamberton, R. W., Morley, S. M., Maguire, P. D. & McLaughlin, J. A. Monitoring laser induced microstructural changes of thin film hydrogenated amorphous carbon (a-CH) using Raman spectroscopy. *Thin Solid Films* **333**, 114-125; doi:10.1016/S0040-6090(98)00848-7 (1998)

15 Mechler, Á. *et al.* Raman spectroscopic and atomic force microscopic study of graphite ablation at 193 and 248 nm. *Appl. Surf. Sci.* **154–155**, 22-28; doi:10.1016/S0169-4332(99)00473-0 (2000)

16 Scott, C. D., Arepalli, S., Nikolaev, P. & Smalley, R. E. Growth mechanisms for single-wall carbon nanotubes in a laser-ablation process. *Appl. Phys. A* **72**, 573-580; doi:10.1007/s003390100761 (2001)

17 Corio, P., Santos, P. S., Pimenta, M. A. & Dresselhaus, M. S. Evolution of the molecular structure of metallic and semiconducting carbon nanotubes under laser irradiation. *Chem. Phys. Lett.* **360**, 557-564; doi:10.1016/S0009-2614(02)00866-7 (2002)
18 Lebedkin, S. *et al.* Single-wall carbon nanotubes with diameters approaching 6 nm obtained by laser vaporization. *Carbon* **40**, 417-423; doi:10.1016/S0008-6223(01)00119-1 (2002)
19 Ueda, T. *et al.* Effect of laser irradiation on carbon nanotube films for NOx gas sensor. *Surf. Coat. Technol.* **202**, 5325-5328; doi:10.1016/j.surfcoat.2008.06.009 (2008)
20 Miyajima, Y., Adikaari, A. A. D. T., Henley, S. J., Shannon, J. M. & Silva, S. R. P. Electrical properties of pulsed UV laser irradiated amorphous carbon. *Appl. Phys. Lett.* **92**, 152104-152104-152103; doi:10.1063/1.2908208 (2008)
21 Lenner, M., Kaplan, A., Huchon, C. & Palmer, R. E. Ultrafast laser ablation of graphite. *Phys. Rev. B: Condens. Matter* **79**, 184105; doi:10.1103/PhysRevB.79.184105 (2009)
22 Cappelli, E., Scilletta, C., Orlando, S., Valentini, V. & Servidori, M. Laser annealing of amorphous carbon films. *Appl. Surf. Sci.* **255**, 5620-5625; doi:10.1016/j.apsusc.2008.10.062 (2009)
23 Sokolov, D. A., Shepperd, K. R. & Orlando, T. M. Formation of Graphene Features from Direct Laser-Induced Reduction of Graphite Oxide. *J. Phys. Chem. Lett.* **1**, 2633-2636; doi:10.1021/jz100790y (2010)
24 Zhang, Y. *et al.* Direct imprinting of microcircuits on graphene oxides film by femtosecond laser reduction. *Nano Today* **5**, 15-20; doi:10.1016/j.nantod.2009.12.009 (2010)
25 Shakerzadeh, M. *et al.* Field emission enhancement and microstructural changes of carbon films by single pulse laser irradiation. *Carbon* **49**, 1018-1024; doi:10.1016/j.carbon.2010.11.010 (2011)
26 Smirnov, V. A. *et al.* Photoreduction of graphite oxide. *High Energ. Chem.* **45**, 57-61; doi:10.1134/s0018143911010176 (2011)
27 Dhar, S. *et al.* A new route to graphene layers by selective laser ablation. *AIP Adv.* **1**, 022109; doi:10.1063/1.3584204 (2011)
28 Xu, N. *et al.* Electrical properties of textured carbon film formed by pulsed laser annealing. *Diamond Relat. Mater.* **23**, 135-139; doi:10.1016/j.diamond.2012.01.016 (2012)
29 Mortazavi, S. Z., Parvin, P. & Reyhani, A. Fabrication of graphene based on Q-switched Nd:YAG laser ablation of graphite target in liquid nitrogen. *Laser Phys. Lett.* **9**, 547; doi:10.7452/lapl.201210033 (2012)
30 Fatt Teoh, H., Tao, Y., Soon Tok, E., Wei Ho, G. & Haur Sow, C. Direct laser-enabled graphene oxide–Reduced graphene oxide layered structures with micropatterning. *J. Appl. Phys.* **112**, 064309; doi:10.1063/1.4752752 (2012)
31 Yung, K. C. *et al.* Laser direct patterning of a reduced-graphene oxide transparent circuit on a graphene oxide thin film. *J. Appl. Phys.* **113**, 244903; doi:10.1063/1.4812233 (2013)
32 Sokolov, D. A., Rouleau, C. M., Geohegan, D. B. & Orlando, T. M. Excimer laser reduction and patterning of graphite oxide. *Carbon* **53**, 81-89; doi:10.1016/j.carbon.2012.10.034 (2013)
33 Petridis, C. *et al.* Post-fabrication, in situ laser reduction of graphene oxide devices. *Appl. Phys. Lett.* **102**, 093115; doi:10.1063/1.4794901 (2013)

34 Chen, H.-Y., Han, D., Tian, Y., Shao, R. & Wei, S. Mask-free and programmable patterning of graphene by ultrafast laser direct writing. *Chem. Phys.* **430**, 13-17; doi:10.1016/j.chemphys.2013.12.005 (2014)

35 Spanò, S. F., Isgrò, G., Russo, P., Fragalà, M. E. & Compagnini, G. Tunable properties of graphene oxide reduced by laser irradiation. *Appl. Phys. A* **117**, 19-23; doi:10.1007/s00339-014-8508-y (2014)

36 Viskadouros, G., Konios, D., Kymakis, E. & Stratakis, E. Direct laser writing of flexible graphene field emitters. *Appl. Phys. Lett.* **105**, 203104; doi:10.1063/1.4902130 (2014)

37 Sahin, R., Simsek, E. & Akturk, S. Nanoscale patterning of graphene through femtosecond laser ablation. *Appl. Phys. Lett.* **104**, 053118; doi:10.1063/1.4864616 (2014)

38 Beltaos, A. *et al.* Femtosecond laser induced periodic surface structures on multi-layer graphene. *J. Appl. Phys.* **116**, 204306; doi:10.1063/1.4902950 (2014)

39 Tite, T. *et al.* Graphene-based textured surface by pulsed laser deposition as a robust platform for surface enhanced Raman scattering applications. *Appl. Phys. Lett.* **104**, 041912; doi:10.1063/1.4863824 (2014)

40 Kumar, I. & Khare, A. Multi- and few-layer graphene on insulating substrate via pulsed laser deposition technique. *Appl. Surf. Sci.* **317**, 1004-1009; doi:10.1016/j.apsusc.2014.08.185 (2014)

41 Xu, S C. *et al.* Direct synthesis of graphene on any nonmetallic substrate based on KrF laser ablation of ordered pyrolytic graphite. *Laser Phys. Lett.* **11**, 096001; doi:10.1088/1612-2011/11/9/096001 (2014)

42 Lin, T. N. *et al.* Laser-ablation production of graphene oxide nanostructures: from ribbons to quantum dots. *Nanoscale* **7**, 2708-2715; doi:10.1039/C4NR05737F (2015)

43 Bulgakova, N. & Bulgakov, A. Pulsed laser ablation of solids: transition from normal vaporization to phase explosion. *Appl. Phys. A* **73**, 199-208; doi:10.1007/s003390000686 (2001)

44 Stagg, B. J. & Charalampopoulos, T. T. Refractive indices of pyrolytic graphite, amorphous carbon, and flame soot in the temperature range 25° to 600°C. *Combust. Flame* **94**, 381-396; doi:10.1016/0010-2180(93)90121-I (1993)

45 ScilabEnterprises (2014). Scilab : Logiciel open source gratuit de calcul numerique, (Windows 7, Version 5.5.0), France. url: http://www.scilab.org
